# Quality of life and comorbidity among older home care clients: role of positive attitudes toward aging

**DOI:** 10.1007/s11136-014-0899-x

**Published:** 2014-12-20

**Authors:** Yukari Yamada, Lukas Merz, Helena Kisvetrova

**Affiliations:** 1 Department of Nursing, Faculty of Health Sciences, Palacky University, Olomouc, Czech Republic; 2 Department of Humanities and Social Sciences, Faculty of Health Sciences, Palacky University, Olomouc, Czech Republic

**Keywords:** Older people, Home care clients, Attitudes to aging, Comorbidity, Quality of life, Additive interaction

## Abstract

**Purpose:**

Comorbidity has a negative impact on quality of life (QoL). This study aimed to investigate whether the impact of comorbidity on QoL is lower in older home care clients with positive attitudes toward aging.

**Methods:**

Totally, 361 older adults aged 50–91 years who were clients of 14 home care agencies in two regions in the Czech Republic gave an in-person interview to research nurses and completed the WHOQOL-BREF, the WHOQOL-OLD, and the Attitudes to Aging Questionnaire. The Charlson comorbidity index was calculated using ICD-10 codes. To address possible interaction between comorbidity and attitudes toward aging for QoL, the presence of additive interaction between comorbidity and attitudes toward aging on QoL was examined by synergy index. All analyses were adjusted by age, gender, education, and living arrangement.

**Results:**

A higher comorbidity index was significantly associated with lower scores of both QoL measures; one index increase was associated with 3.7 [95 % confidence interval (CI) 1.5: 5.9] decreases in generic QoL and 3.6 (95 % CI 1.3: 5.9) decreases in older-specific QoL. In stratified analyses by attitudes toward aging, comorbidity showed no association with QoL among those with positive attitudes, while it was significantly associated with low QoL in those without positive attitudes. The presence of additive interactions between comorbidity and less than positive attitudes on falling in low QoL was clearly suggested.

**Conclusions:**

The negative impact of comorbidity on QoL might be mitigated by promoting a positive self-perception of aging in older people.

**Electronic supplementary material:**

The online version of this article (doi:10.1007/s11136-014-0899-x) contains supplementary material, which is available to authorized users.

## Introduction

Comorbidity is common among older home care clients. The impact of comorbidity operationalized as the count of comorbid conditions or the weighted comorbidity index on various health outcomes has been widely documented [1]. One of the most important health outcomes that can be influenced by comorbidity is quality of life (QoL) [2–7]. QoL, a product of the individual’s subjective view of illness [8], is known to predict mortality and hospitalization [9–13]. Improving QoL is an important health care topic in an aging society [14]; therefore, addressing any modifiable factor that can prevent the negative impact of comorbidity on QoL would benefit health policies.

One of the factors known to be associated with QoL is one’s attitudes toward aging or self-perceptions of aging [15–19]. Attitudes toward aging is a framework for assessing one’s own aging experiences as well as one’s attitudes toward older people [20], and it has been suggested that older people who hold a positive view on aging have good psychological resources even in old age [18]. In fact, a growing body of empirical data has demonstrated that beliefs about aging are associated with health behaviors and outcomes [21–24]. A possible causal pathway directed from attitudes toward aging to health outcomes has been discussed based on some findings that older adults with low expectations to aging were less likely to lead a healthy life style [25, 26], seek health care for age-associated conditions [27], and were more likely to have a heightened cardiovascular response to stress [28], while the opposite direction of the causal pathway can be also possible; poor health affects a negative attitude toward aging [22, 29].

Given that the growth, development, and positive change in attitudes toward and perceptions of aging are possible and commonplace among older adults [29], promoting positive attitudes toward aging may modify the negative impact of comorbidity on QoL, although this possibility has not been investigated till date. The objective of the present study was to examine an effect modification of positive attitudes toward aging in the association between comorbidity and poor QoL.

## Methods

### Participants and data collection

Home care clients of 14 home care agencies in two regions (Olomouc, Zlin) in the Czech Republic were invited to participate in our study between May and November 2012. Eligibility criteria included age ≥50 years and the absence of cognitive impairments. The age criteria was applied, because it was used in a validity study of the WHOQOL-OLD in the Czech Republic [30] and also it is the age that is considered as an important milestone in psychology that people would experience changing their attitudes to world and self [31]. The research nurses visited older people’s home and obtained informed consent for participating in our study. The participants were then asked to complete questionnaires. The research nurses ensured that the clients fully understood the questions in the questionnaire and physically helped the clients to complete the questionnaire if required. Registered diagnoses of the participants as coded in the International Classification of Diseases version 10 (ICD-10) were obtained through medical records registered at the home care agencies. Information on age, gender, education, and living arrangement was also recorded. The data collection continued to reach a minimum of 300 participants. The final sample included 361 older adults. The study was approved by the Ethics Committee of the Faculty of Health Sciences, Palacky University in Olomouc.

### Measurements

Three authorized Czech versions of questionnaires developed by the World Health Organization [30] were used; WHOQOL-BREF, WHOQOL-OLD, and Attitudes to Aging Questionnaire (AAQ).

#### Measurements of QoL

The WHOQOL-BREF and the WHOQOL-OLD were used to measure generic and older-specific QoL, respectively. Both measures include 24 items rated on a five-point Likert-type response scale, and higher scores indicate a higher QoL. The WHOQOL-BREF [32] covers four domains: physical (7 items), psychological (6 items), social relationships (3 items), and environmental (8 items). Although it was not developed specifically for an older population, its applicability in older adult populations has been reported [33, 34]. Cronbach’s alpha coefficient values for the WHOQOL-BREF total score was 0.92 in our study. The WHOQOL-OLD [35] covers six domains, including sensory abilities; autonomy; past, present, and future activities; social participation; death and dying; and intimacy, each of which has 4 items. The internal consistency reliability was 0.91 in our study. Since our focus was to clarify how comorbidity has its impact on one’s QoL, this study did not use domain scores, but used total scores of the WHOQOL-BREF and the WHOQOL-OLD as *generic QoL* and *older-specific QoL*. Both measures were transformed to a range of 0–100 for description and comparison purposes.

#### Measurement of attitudes toward aging

The participants’ perspective of their own aging process was measured using the AAQ [36]. The AAQ is a 24-item questionnaire comprising three factors encompassing psychological losses, physical change, and psychological growth, each of which has 8 items. The total AAQ score ranges from 24 to 120, and a higher score indicates more positive attitudes toward one’s own aging process. The subscale structure of the AAQ has been established in 15 countries using classical and modern psychometric methods [15, 16, 36–38]. The internal consistency reliability was 0.83 in our study. The total AAQ was both used as a numerical value and was categorized into tertiles; the total AAQ score of 69 and below was in the lowest tertile, 70–79 was in the middle tertile, and 80 and over was in the highest tertile. Positive attitudes to aging was defined the highest tertile in our study.

#### Measurement of comorbidity

The Charlson comorbidity index (CCI) [39] was used as a weighted comorbidity index. The original CCI is a list of 17 conditions, each of which has a weight from one to six, which was developed based on relative risk estimates of morality using clinical data [39, 40]. A Charlson score was calculated for each participant by adding the weights assigned to each disease diagnosed in that patient (e.g., congestive heart failure [1] + lymphoma [2] = Charlson score [3]). The Charlson score was prospectively consolidated into four previously defined groups known as the CCI: 0 points (none), 1.2 points (low), 3.4 points (moderate), and 5 points (high) as originally described [39]. In our study, moderate and high were merged since there were few people (5 people) in the high index. The CCI was used both as a continuous variable and as the categorical variable.

### Analyses

The means of QoL measures according to the various characteristics of the participants were described and one-way analyses of variances were conducted to compare the means. When the characteristics represent ordered group, age group and education, linear trends were tested.

Generalized linear models were applied to estimate means of QoL measures according to categorized comorbidity and attitudes to aging as well as estimate regression coefficients of both categorical and numerical variables, adjusted for age (numerical), gender, education (primary and vocational vs. secondary and university), and living arrangements (with partner or family vs. alone).

In order to address a possible interaction between comorbidity and attitudes toward aging in terms of the risk of poor QoL, we applied two different strategies. First, the above-mentioned generalized linear models were stratified by attitudes toward aging in order to examine differences in the associations of comorbidity with QoL measures between those with positive attitudes toward aging and those without. Second, we made a combined exposure variable of comorbidity (none vs. others) and attitudes toward aging (positive vs. others) and defined participants whose QoL score was less than the median score on each QoL measure (i.e., <59.0 for generic QoL and <61.5 for older-specific QoL) as *low QoL.* Then, odds ratios for the combined variable were calculated using logistic regression models with *low QoL* as a dependent variable, adjusted for the same covariates as in the generalized linear models (age, gender, education, and living arrangements).

We used the synergy index (SI) to examine the magnitude of the additive interaction [41]. The SI is interpreted as excess risk from exposure to both factors when there is an interaction relative to the risk from exposure without interaction: SI = [(odds ratio for joint exposure to both risk factors − 1)/[(odds ratio for one risk factor − 1) + (odds ratio for other risk factors − 1)] [41]. In the absence of an interaction effect, the SI equals 1. The 95 % confidence interval (CI) was computed using the recommended formula [42]. To examine the robustness of findings, sensitivity analyses with an alternative definition of *low QoL* as the lowest 30 % (i.e., <51.6 for generic QoL and <54.1 for older-specific QoL) on each QoL measure were conducted. All data analyses were performed using IBM SPSS version 22.

## Results

The mean age of participants was 77.3 years (median 78, interquartile range 14). Table 1 indicates the means of QoL measures according to the participants’ characteristics. Younger participants had higher QoL measures than older participants. Gender was not associated with older-specific QoL, while women were more likely to have a higher generic QoL than men. Home care clients with higher education and those living with someone else had higher QoL measures compared with their counterparts.

**Table 1:** Means and standard deviations of QoL scores according to characteristics of home care users (*N* = 361)

Characteristics	*N* (%)	Generic QOL			Older-specific QOL		
		Mean	SD	*P* value*	mean	SD	*P* value*
*Overall*							
Gender	361 (100)	58.2	14.0		61.4	14.7	
Female	239 (66)	59.3	13.4	0.036	61.7	14.9	0.579
Male	122 (34)	56.1	14.9		60.8	14.4	
Age							
<69	76 (21)	60.8	15.9	0.018	64.8	15.6	0.004
70–79	122 (34)	59.2	14.0		62.5	15.7	
≥80	163 (45)	56.2	12.8		58.9	13.1	
Education							
Primary	111 (31)	56.1	12.5	0.038	58.9	13.3	0.014
Vocational	108 (30)	57.6	14.4		60.7	15.0	
Secondary	108 (30)	60.0	13.3		63.3	13.7	
University	34 (9)	61.2	18.1		65.3	19.8	
Living arrangements							
With partner only	138 (38)	60.9	15.3	0.006	65.1	15.2	<0.001
With other family	93 (26)	58.0	12.9		60.6	14.6	
Alone	130 (36)	55.5	12.8		57.9	13.3	

*SD* standard deviation

* One-way analysis of variance for a quantitative dependent variable (generic QoL and older-specific QoL) by independent variables (gender, age group, education, and living arrangement). *P* values for age group and education were tested by linear trend of the dependent variables

Table 2 demonstrates the associations of comorbidity and attitudes toward aging with QoL measures, separately. Participants with a higher comorbidity index had lower QoL measures; one index increase was associated with 3.7 decreases in generic QoL and 3.6 decreases in older-specific QoL. Participants who had more negative attitudes toward aging had lower QoL measures; one score increase was associated with 0.8 increases in generic QoL and 0.9 increases in older-specific QoL.

**Table 2:** Estimated means of QoL measures according to comorbidity and attitudes to aging (*N* = 361)

Comorbidity/attitudes to aging	*N*	Generic QoL			Older-specific QoL		
		Mean (SE)	Coefficient (95 % CI)	*P* value	Mean (SE)	Coefficient (95 % CI)	*P* value
Comorbidity							
None	176	60.6 (1.1)	(Reference)	–	64.4 (1.1)	(Reference)	–
Low	159	56.5 (1.1)	−4.2 (−7.0:−1.3)	0.004	59.6 (1.1)	−4.8 (−7.8:−1.8)	0.002
Moderate and high	26	54.1 (2.6)	−6.6 (−12.1:−1.1)	0.019	59.4 (2.7)	−5.0 (−10.7: 0.8)	0.089
CCI (continuous)	361		−3.7 (−5.9:−1.5)	0.001		−3.6 (−5.9:−1.3)	0.003
Attitudes to aging							
Highest tertile	112	69.1 (1.0)	(Reference)	–	73.6 (1.1)	(Reference)	–
Middle tertile	118	58.3 (1.0)	−10.8 (−13.6:−8.0)	<0.001	61.9 (1.0)	−11.7 (−14.5:− 8.8)	<0.001
Lowest tertile	131	48.5 (1.0)	−20.6 (−23.3:−17.9)	<0.001	51.3 (1.0)	−22.3 (−25.1:−19.5)	<0.001
AAQ (continuous)	361		0.8 (0.7: 0.9)	<0.001		0.9 (0.8: 1.0)	<0.001

Generalized linear models adjusted for age, gender, education, and living arrangements

*SE* standard error, *CI* confidence interval, *CCI* Charlson comorbidity index (a higher index indicates a severer comorbidity), *AAQ* Attitudes to Aging Questionnaire (a higher score indicates more positive attitudes toward aging)

Table 3 shows the associations between comorbidity and QoL measures, stratified by dichotomized attitudes toward aging (i.e., those with positive attitudes to aging and those without positive attitudes to aging). Comorbidity showed no association with QoL measures among participants with positive attitudes toward aging. This finding opposed that for participants without positive attitudes toward aging, comorbidity was significantly associated with lower QoL measures; one index increase was associated with 3.1 decreases in both generic and older-specific QoL measures.

**Table 3:** Estimated means of QoL measures according to comorbidity stratified by attitudes to aging (*N* = 361)

Comorbidity	*N*	Generic QoL			Older-specific QoL		
		Mean (SE)	Coefficient (95 % CI)	*P* value	Mean (SE)	Coefficient (95 % CI)	*P* value
Among those with positive attitudes							
None	67	68.7 (1.3)	(Reference)	–	72.2 (1.3)	(Reference)	–
Low	40	70.1 (1.7)	1.4 (−2.6: 5.5)	0.488	75.5 (1.6)	3.3 (−0.7: 7.2)	0.108
Moderate and high	5	67.2 (4.6)	−1.5 (−11.0: 7.9)	0.748	68.5 (4.5)	−3.7 (−13.0: 5.5)	0.430
CCI (continuous)	112		0.5 (−2.8: 3.8)	0.753		0.7 (−2.0: 3.4)	0.598
Among those without positive attitudes							
None	109	55.2 (1.2)	(Reference)	–	59.2 (1.2)	(Reference)	–
Low	119	51.3 (1.1)	−3.9 (−6.9:−0.9)	0.012	53.8 (1.2)	−5.5 (−8.6:−2.3)	0.001
Moderate and high	21	50.3 (2.6)	−4.9 (−10.4: 0.54)	0.077	56.7 (2.6)	−2.5 (−8.2: 3.1)	0.377
CCI (continuous)	249		−3.1 (−5.4:−0.8)	0.009		−3.1 (−5.5: 0.7)	0.013

*SE* standard error, *CI* confidence interval, *CCI* Charlson comorbidity index (a higher index indicates a severer comorbidity)

Generalized linear models adjusted for age, gender, education and living arrangements

Table 4 presented the results of analyses with the combined variables. Home care clients with one or more comorbidity and without positive attitudes toward aging had a 11-fold higher risk of generic *low QoL* and a 16-fold higher risk of older-specific *low QoL* compared with those without comorbidity and with positive attitudes. The synergy indexes indicated the presence of additive interactions between comorbidity and attitudes toward aging for the *low QoL measures*; the joint associations were two times larger than those expected when summing up the individual risks of comorbidity and less than positive attitudes in both generic and older-specific QoL measures.

**Table 4:** Adjusted odds ratios of combined variable of comorbidity and attitudes to aging for *Low QoL*

	*N*	Generic QoL OR (95 % CI)	Older-specific QoL OR (95 % CI)
Positive attitude + no comorbidity (ref.)	67	1.0	1.0
Positive attitude + comorbidity	45	0.6 (0.2: 1.9)	1.1 (0.4: 3.3)
Not positive attitude + no comorbidity	109	5.8 (2.7: 12.2)	8.0 (3.5: 18.0)
Not positive attitude + comorbidity	140	11.2 (5.3: 23.3)	16.2 (7.2: 36.5)
Synergy index^a^		2.3 (1.1: 4.6)	2.1 (1.1: 4.0)

*Low QoL* was defined as a QoL score being less than the median score on each QoL measure for all participants

Logistic regression adjusted for age, gender, education, and living arrangements

*OR* odds ratio, *CI* confidence interval, *ref* reference

^a^In the absence of an additive interaction effect, the synergy index equals 1

Sensitivity analyses with an alternative definition of *low QoL* as the lowest 30 % revealed the same directions of the associations in all analyses. The magnitude of the associations did not substantially change, but the synergy index for older-specific QoL had a wider confidence interval (data not shown).

## Discussion

In this cross-sectional study on home care clients in the Czech Republic, comorbidity was negatively associated with QoL, but this association was not observed among those having positive attitudes toward aging. Furthermore, comorbidity and not positive attitudes toward aging had a synergic effect on an increased risk of falling in poor QoL. The overall findings in our study may support our hypothesis that the negative effect of comorbidity on QoL might be mitigated by the presence of positive attitudes toward aging.

The findings of this study are fairly consistent with those of previous studies demonstrating QoL as a function of comorbidity [2–7] as well as attitudes toward aging [15–18]. Possible mediating roles of attitudes toward aging in the relationship between a poor health condition and QoL have also been suggested in some studies [15, 19]. Attitudes toward aging were suggested to be a mediator of relationships between the subjective health of older adults and QoL in 20 countries [15] and those between cognitive and physical function and QOL of older adults with dementia [19]. To the best of our knowledge, this is the first study to demonstrate that attitudes toward aging may modify the association between comorbidity and QoL.

Our findings are primarily consistent across generic and older-specific QoL measures; both had negative associations with comorbidity, and attitudes to aging were interacted in both associations. This may be not surprising given a high correlation between generic and older-specific QoL [35]. We have found, however, that women had a higher generic QoL than men, while there was no gender difference observed in older-specific QoL. A closer look at data revealed that this was clearly due to a higher score for the domain of social relationships in generic QoL among women. The evidence on gender differences in QoL in general population samples has been mixed [43]. Our findings may indicate that older-specific QoL is comparable between men and women in home care clients, but generic QoL is not due to a gender difference in social relationships.

Assuming that the negative effect of comorbidity on QoL is mitigated by the presence of positive attitudes toward aging, the findings of this study imply a possibility that the promotion of positive attitudes toward aging will alleviate the negative impact of illness on the QoL of older adults. Possible ways of improving attitudes toward aging in older adults have been discussed. Theoretical analyses recommend a change in age stereotypes in the media to be more active, for example [44], although an experimental study revealed a rather negative influence of the activation of positive age stereotypes [45]. Studies on successful aging showed modifiable factors at the age of 50 years, such as smoking and regular exercise, and coping mechanisms influencing the quality of subjective and objective aging at the age of 70–80 years [46]. Therefore, promoting health behavior may improve the attitudes toward aging in older adults.

There are some limitations to this study. First, although we speculated a possible protective role of attitudes toward aging against the negative impact of comorbidity on QoL, the cross-sectional study design did not allow us to draw firm conclusions. A further longitudinal study is needed to confirm the direction of the associations and determine whether people with positive attitudes toward aging are less likely to diminish their QoL later in life, even with their comorbidity. Second, the sampling was not randomized; therefore, the participants in the study may not adequately represent adult home care clients without cognitive impairments. Moreover, given this study took place in the Czech Republic and focuses on home care clients in limited regions, generalizability of the findings to other countries that may or may not have a similar model of home care cannot be guaranteed. However, a comparison of the WHOQOL-BREF scores of our sample with those of an international large sample of people aged ≥60 years [47] shows that the scores of our sample had almost identical variances and slightly lower means across the four domains, with the greatest difference being observed in the physical domain. This comparison may support that our sample was not divergent from the expectations from older home care clients in other countries. Thirdly, there might be measurement bias in the comorbidities with a lack of details available regarding severity and duration of the comorbidities. Specifically, it is possible that the main result (i.e., comorbidity has a greater negative impact on subjects with no positive attitudes toward aging) was due to the fact that people with positive attitudes have actually less severe comorbidity (and hence more positive attitudes). However, a closer look at the mean comorbidity scores (Charlson comorbidity score) and types of comorbidities between those with positive attitudes and those with less than positive attitudes (see supplementary data) seems to indicate a proximity rather than a differential severity of comorbidities between the groups.

## Conclusions

The negative impact of comorbidity on QoL might be mitigated by promoting a positive self-perception of aging in older people.

## Electronic supplementary material

Below is the link to the electronic supplementary material.

Supplementary material 1 (DOCX 18 kb)
