# Supplementary material for: Quality of life and comorbidity among older home care clients: role of positive attitudes toward aging
Source: Qual Life Res. 2014 Dec 20;24(7):1661–7. doi: 10.1007/s11136-014-0899-x (PMC4483185; doi:10.1007/s11136-014-0899-x)
Supplement: Supplementary file 1 — Supplementary material 1 (DOCX 18 kb) [file 11136_2014_899_MOESM1_ESM.docx]

**Article Title:** Quality of life and comorbidity among older home care clients: role of positive attitudes toward aging

**Journal Name:** Quality of Life Research

**Author Names:** Yukari Yamada, Lukas Merz, Helena Kisvetrova

**Affiliation and e-mail address of the corresponding author:**

Yukari Yamada

Faculty of Health Sciences, Palacky University in Olomouc

yukari.yamada@upol.cz

Comorbidities distributions according to attitudes to ageing among people with comorbidity (n=185)

|  | Positive Attitude (N=45) | Not Positive Attitude(N=140) | P value |
| --- | --- | --- | --- |
| Myocardial Infarction, % | 2.2 | 5.7 | 0.690 |
| Congestive Heart Failure, % | 2.2 | 5.7 | 0.690 |
| Peripheral Vascular Disease, % | 0.0 | 4.3 | 0.338 |
| Cerebrovascular Disease, % | 15.6 | 33.6 | 0.024 |
| Dementia, % | 4.4 | 6.4 | 1.000 |
| COPD, % | 13.3 | 15.0 | 1.000 |
| Connective Tissue Disease, % | 13.3 | 15.0 | 1.000 |
| Peptic Ulcer Disease, % | 6.7 | 0.7 | 0.045 |
| Mild Liver Disease, % | 0.0 | 0.0 | - |
| Diabetes Without Complications, % | 33.3 | 35.7 | 0.858 |
| Diabetes With Chronic Complications, % | 15.6 | 6.4 | 0.070 |
| Hemiplegia Or Paraplegia, % | 4.4 | 3.6 | 0.678 |
| Renal Disease, % | 6.7 | 2.1 | 0.156 |
| Any Malignancy, including Leukemia and Lymphoma, % | 2.2 | 5.7 | 0.690 |
| Moderate Or Severe Liver Disease. , % | 2.2 | 2.1 | 1.000 |
| Metastatic Solid Tumor, % | 0.0 | 1.4 | 1.000 |
| AIDS/HIV , % | 0.0 | 0.0 | - |
| CCI, mean(SD) | 1.44 (0.76) | 1.69 (1.08) | 0.153 |

P values are obtained from chi square tests for the type of comorbidities and t-tests for CCI.
